# Drugless peptide-based nanohybrids alleviate diabetic retinopathy by suppressing microglial activation and endothelial inflammation

**DOI:** 10.7150/thno.102775

**Published:** 2025-03-03

**Authors:** Mei Du, Xiao Zhao, Miao Guo, Xiaoyu Wang, Yutian Zhang, Linshan Yang, Sixia Liu, Liya Sun, Mengyu Liao, Xue Dong, Yi Lei, Yumeng Zhao, Shuqi Liang, Xiaohong Wang, Caiyun You, Hong Yang, Hua Yan

**Affiliations:** 1 Laboratory of Molecular Ophthalmology, Tianjin Medical University, Tianjin 300070, China.; 2 Department of Ophthalmology, Tianjin Medical University General Hospital, Ministry of Education International Joint Laboratory of Ocular Diseases, China-UK “Belt and Road” Ophthalmology Joint Laboratory, Tianjin Key Laboratory of Ocular Trauma, Tianjin Institute of Eye Health and Eye Diseases, Tianjin 300052, China.; 3 Tianjin Key Laboratory of Inflammatory Biology, Department of Pharmacology, School of Basic Medical Sciences, Tianjin Medical University, Tianjin, China.; 4 The Province and Ministry Co-Sponsored Collaborative Innovation Center for Medical Epigenetics, Intensive Care Unit of the Second Hospital, Tianjin Medical University Tianjin, China.

**Keywords:** diabetic retinopathy, bioactive nanoparticles, endothelial inflammation, microglia activation, Toll-like receptors

## Abstract

**Background:** Diabetic retinopathy (DR) is a vision-threatening microvascular complication of diabetes mellitus. Chronic inflammation and endothelial dysfunction are critical factors in the disease's pathogenesis. Consequently, interventions developed to reduce retinal inflammation are anticipated to be beneficial for both the prevention and treatment of DR. In the present study, we developed a unique class of drugless peptide-based nanohybrids with potent anti-inflammatory activities and investigated their therapeutic efficacy for treating DR in an oxygen-induced retinopathy (OIR) mouse model and a streptozotocin (STZ)-induced diabetic mouse model.

**Methods:** Hexapeptides were applied to modify gold nanoparticles to form the drugless peptide-based nanohybrids (P12). We then examined the physicochemical properties and anti-inflammatory activities of P12 in HUVECs and BV2 cells and identified the critical amino acids for this novel bioactivity. The intravitreal and retro-orbital injections were applied to determine the optimal retinal delivery route for P12*.* The therapeutic efficacy of P12 in treating DR were investigated using both the OIR model and STZ-induced diabetic model. Through immunohistochemistry and flow cytometry analyses, we identified the major cells that internalize P12 in the retina. Furthermore, *in vitro* experiments were used to explore the underlying molecular mechanisms for the anti-inflammatory activities of P12.

**Results:** We found that P12 exhibited potent anti-inflammatory effects in both HUVECs and BV2 cells. In addition, P12 can be efficiently delivered to the retina via intravitreal injection. Intravitreally injected P12 significantly improved early DR symptoms including vascular leakage and pericyte loss in STZ-induced diabetic mice. It also suppressed pathological neovascularization and retinal hemorrhage in OIR mice. Importantly, we found that intravitreally injected P12 was mainly taken up by microglial and endothelial cells, leading to reduced retinal endothelium inflammation and microglial activation in DR animal models. Mechanistic studies revealed that P12 potently inhibited several TLR4 downstream signaling pathways, such as NF-κB, JNK, and P38 MAPK, in both endothelial and microglial cells. This effect is due to the capacity of P12 in blocking the endosomal acidification process that governs the endosomal TLR signaling transduction.

**Conclusions:** Our findings suggest that local injection of properly designed, drugless, peptide-based nanohybrids can serve as a safe and effective anti-inflammatory nanomedicine for treating DR.

## Introduction

Diabetic retinopathy (DR) is a common microvascular complication of diabetes mellitus (DM) and a leading cause of visual impairment and blindness in adults 1. The rising adoption of Western dietary habits, combined with an aging population, has led to a significant increase in the incidence of diabetes and its associated complications. By 2045, DM is estimated to affect 783.2 million people 2, with 35% expected to develop some form of retinopathy 3. DR is categorized into two stages: early nonproliferative DR (NPDR) and advanced proliferative DR (PDR). NPDR is characterized by basement membrane thickening, pericyte loss, microaneurysm formation, and disruption of the blood-retinal barrier (BRB) 4. Hypoxia and retinal ischemia in NPDR drive the development of retinal neovascularization, a hallmark of PDR 4. These newly formed vessels are immature and leaky, resulting in retinal edema, scar formation, and retinal detachment, which are the primary causes of vision loss in patients with DR 4. First-line therapies for DR include intraocular steroid injection or anti-vascular endothelial growth factor (VEGF) agents. However, steroid use can lead to steroid-induced glaucoma and cataracts, whereas long-term anti-VEGF injections may lead to retinal neurodegeneration and infectious endophthalmitis 5, 6. Therefore, the development of novel therapeutic approaches for DR is urgently required.

Hyperglycemia is the primary risk factor for DR, but even with good glycemic control, patients with DM are still at risk of developing retinopathy, suggesting the involvement of additional risk factors in the disease 7, 8. Accumulating evidence from preclinical and clinical studies supports the involvement of inflammation in DR 9. Pro-inflammatory molecules, such as IL-1β, IL-12, and IL-6, are elevated in the aqueous humor and vitreous fluid of patients with DR 10. Retinal capillary endothelial cells (ECs), which line the vasculature of the inner retina and form the inner BRB, are considered key players in DR pathogenesis. Studies have shown that the upregulation of inflammatory cytokines and adhesion molecules promotes BRB breakdown by inducing endothelial dysfunction and leukostasis. This aggravates retinal hypoxia and promotes pathological neovascularization 11. In contrast, the inhibition of the inflammatory cascade significantly mitigates the early morphological signs of DR in diabetic animal models 12.

Microglia are resident macrophages of the retina that trigger pro-inflammatory processes upon activation, contributing to several neurodegenerative diseases, including DR 13. Uncontrolled microglial activation has been documented in both DR animal models and patients with DR 13. In rodents, retinal microglia become activated as early as 1 month after diabetes induction, whereas in patients, microglial activation is observed at different stages of DR 13. Overactivated microglia produce various inflammatory cytokines, chemokines, and adhesion molecules that contribute to retinal vascular damage and neurotoxicity 13. Diverse signaling cascades drive these pathogenic processes, with Toll-like receptors (TLRs) and their downstream signaling pathways playing a significant role 14. TLR4 is expressed in different retinal cell types, including microglia and vascular endothelial cells 15, 16. Accumulating evidence supports the significant role of TLR4 in DR pathogenesis 17-22. In the diabetic retina, hyperglycemia and overproduction of reactive oxygen species may activate TLR4 and its downstream nuclear factor kappa B (NF-κB) pathway, leading to the increased expression of pro-inflammatory genes 17. Additionally, pharmacological inhibition or genetic deletion of TLR4 can reduce retinal inflammation and vascular damage in diabetic animal models, suggesting that TLR4-targeted intervention may be a promising therapeutic strategy for DR 18-22.

In addition to conventional therapies, nanomaterials have shown great potential as efficient drug delivery systems for the treatment of DR. These nanomaterials have good biocompatibility and can improve the bioavailability of drugs by enhancing their penetration through the BRB. For example, lipid-based nanoparticles have been used to deliver anti-inflammatory drugs and genes to the retina 23, 24. Polymeric nanoparticles, such as poly (lactic-co-glycolic acid), have been used to formulate bevacizumab for retinal drug delivery 25. These nanomaterials can protect the loaded drugs from degradation, facilitate sustained drug release, and allow specific cell targeting. In contrast to these approaches, which use nanomaterials as drug carriers, we previously developed a simple, bioactive nanoparticle (P12) that could mitigate multiple TLR signaling pathways and reduce the production of pro-inflammatory cytokines in macrophages without carrying any drug 26, 27. P12 is composed of gold nanoparticles coated with a hexapeptide (CLPFFD). However, whether P12 regulates TLR4 signaling in ECs and microglia during DR treatment remains unclear.

In this study, we investigated the therapeutic efficacy of peptide-gold nanoparticle (GNP) hybrids in the treatment of DR using streptozotocin (STZ)-induced diabetic and oxygen-induced retinopathy (OIR) mouse models. By administering P12 locally into the vitreous cavity, we observed a significant reduction in retinal inflammation and vascular impairment, including vascular leakage, pericyte loss, and pathological neovascularization, in STZ and OIR mouse models. Moreover, intravitreally injected P12 was primarily taken up by retinal microglial and endothelial cells, leading to reduced microglial activation and endothelial dysfunction, partly through the modulation of the TLR4 signaling pathway from the endosomes. This study highlights the potential of nanoparticle-based TLR inhibitors as next-generation anti-inflammatory therapeutics for DR.

## Results

### Screening for peptide-GNP hybrids with anti-inflammatory effects on both retinal microglia and endothelial cells

**Figure 1:**
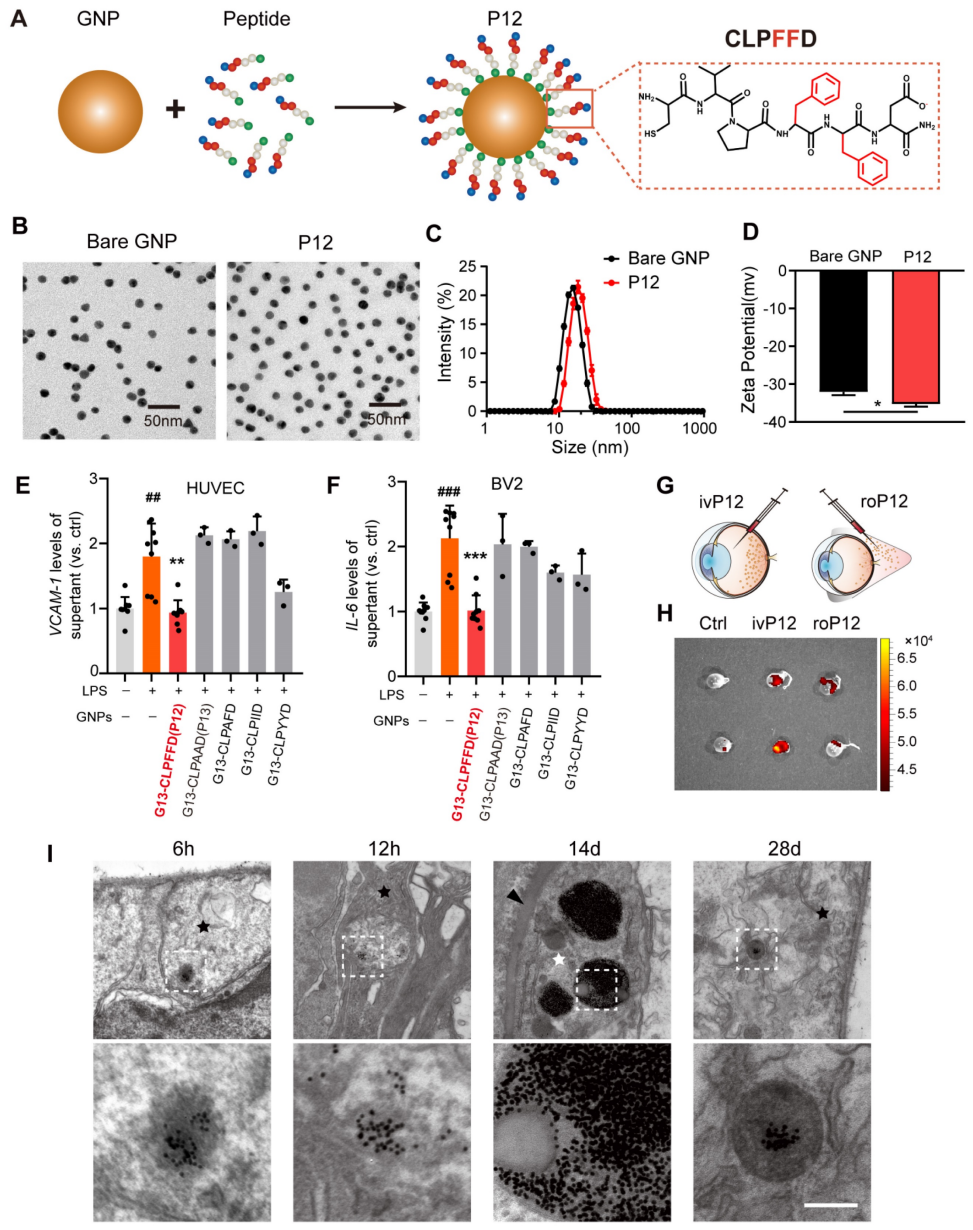
** Screening of anti-inflammatory nanoparticles in microglia and endothelial cells.** (A) The construction of the peptide-GNP hybrids P12. (B) TEM image of Bare GNP and P12. Scale bar = 50 nm. (C) The size distribution of the bare GNP and P12 measured by DLS. (D) The zeta-potential of the bare GNP and P12. N ≥ 3, *p < 0.05. (E, F) ELISA measurement of VCAM-1 and IL-6 levels in the supernatant of HUVEC (E) and BV2 (F) cells with various peptide-GNP hybrids treatment. (G, H) Intravitreal vs. retro-obital delivery of Cy5-labeled P12. Fluorescence of the eyeballs was imaged at 12 h after indicated injection. (I) TEM images showing the distribution of P12 in mouse retina at 6 h, 12 h, 14 d, and 28 d after intravitreal injection. P12 was found in nerve fiber layer (black star) at 6 h,12 h and even 28 d after injection, and was found in pericyte (white star) surrounding the basement layer (black arrowhead) of microvascular in the retina at 14 d after injection. Scale bar = 200 nm.

DR is a microvascular complication characterized by inflammatory reactions in which endothelial dysfunction and uncontrolled microglial activation play central roles 28. To identify bioactive nanoparticles with anti-inflammatory effects on both retinal microglia and endothelial cells, we examined P12 developed in our previous studies 29. P12 is composed of a GNP core with a diameter of 13 nm, which was modified with the CLPFFD hexapeptide **(Figure 1A)**. Peptide modification significantly enhanced the physiological stability of the GNPs and imparted new bioactivity** (Figure S1)**. The physicochemical properties of P12 were also investigated. Transmission electron microscopy (TEM) images of bare GNP and P12 showed similar, uniform spherical structures **(Figure 1B)**. Their size distribution was analyzed using dynamic light scattering, which showed that the hydrodynamic diameter of P12 was 18.5±0.2 nm, approximately 3.4 nm larger than that of bare GNPs (15.1±0.3 nm) **(Figure 1C)**, indicating the successful peptide conjugation to GNPs. The zeta potential of P12 was -35.3±1.2 mV, which was slightly more negative than that of bare GNPs (-32.1±1.5 mV) **(Figure 1D)**.

Next, we examined the anti-inflammatory effects of these peptide-GNP hybrids on HUVECs and BV2 cells. In HUVECs, P12 significantly reduced the expression of adhesion molecules ICAM-1 and VCAM-1 induced by lipopolysaccharide (LPS) **(Figure 1E, Figure S2A)**. In BV2 cells, P12 markedly downregulated LPS-induced IL-6 and MCP-1 expression **(Figure 1F, Figure S2B).** We found that the effective region of “FF” in the peptide sequence contributed to these novel effects. Mutation of “FF” to “AA” (P13), “AF,” “II,” and “YY” diminished the inhibitory activities of the nanohybrid on HUVEC and BV2 activation **(Figure 1E-F, Figure S2A-B)**.

Next, we compared the retinal delivery efficiency of P12 by intravitreal and retro-orbital injections. 6 h after injection, the fluorescent signals of Cy5-labeled P12 were significantly higher in intravitreal-injected eyes than in retro-orbital-injected eyes, suggesting that intravitreal injection was more efficient in delivering P12 to the retina **(Figure 1G-H)**. We then analyzed the dynamics of the retinal distribution of P12 using TEM. 6 h after intravitreal injection, the nanoparticles were observed in the superficial layer of the retina. Over time, the nanoparticles started to appear in the deep retina, such as the nerve fibers, at 12 h post-injection. Interestingly, 14 days after injection, we observed an abundant accumulation of nanoparticles in the pericytes surrounding the microvascular wall. By 28 days post-injection, nanoparticles were still present in the vesicles of the superficial layer of the retina **(Figure 1I)**. These observations suggest that intravitreal injection of P12 could be retained in the retina and taken up by different retinal cells.

### P12 ameliorated retinal vascular leakage and retinal inflammation in STZ-induced diabetic mice

**Figure 2:**
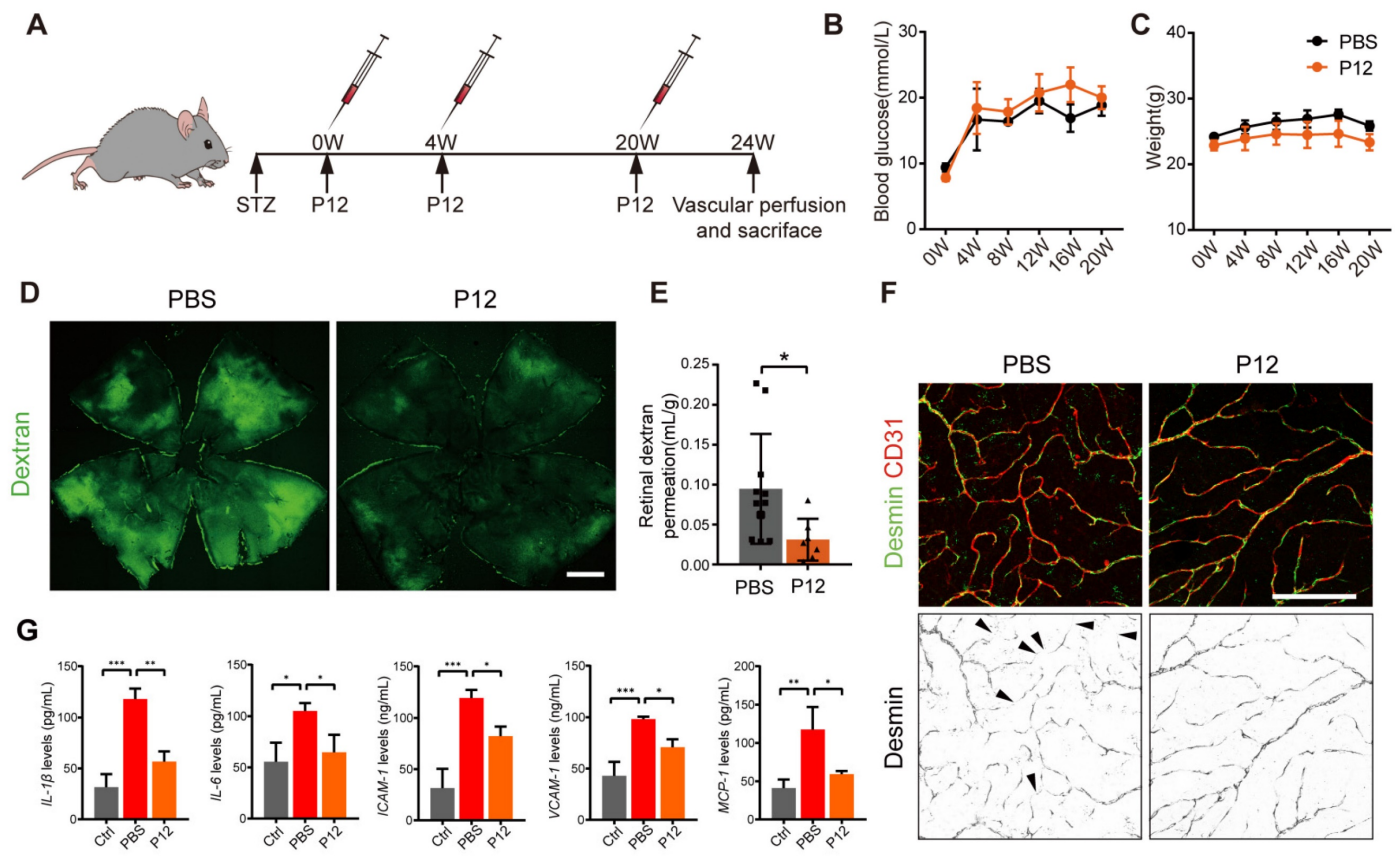
** ivP12 ameliorated retinal vascular leakage and retinal inflammation in STZ-induced diabetic mice.** (A) Schematic depiction of the induction of STZ-induced diabetic mouse model. (B, C) Measurement of blood glucose levels (B) and body weight (C) of PBS and P12 treated mice for 20 weeks post-STZ treatment. (D) Retinal vascular permeability was assessed by FITC-dextran assay in PBS and P12 treated mice at 24 weeks post-STZ injection. Scale bar=500 μm. (E) Quantification of FITC-dextran permeation in PBS and P12 treated mice. (F) Pericyte coverage of retinal vessels was assessed by immunostaining of desmin in retinal flatmounts from PBS and P12 treated mice at 24 weeks post-STZ injection. The black arrows denote the regions of the retinal vessels where pericytes were lost. Scale bar=200 μm. (G) The protein levels of the inflammatory cytokines and adhesion molecules in retina samples of STZ mice with or without P12 treatment.

To investigate the efficacy of intravitreal injection of P12 (ivP12) in treating DR, we utilized STZ-induced diabetic mice as they develop early retinal vascular abnormalities in NPDR 30. P12 was intravitreally injected into the mice at 0, 4, and 20 weeks after STZ induction **(Figure 2A)**. We monitored the blood glucose levels and body weight changes in STZ mice at various time points for up to 20 weeks. IvP12 did not affect these parameters during the 20-week observation period **(Figure 2B-C)**. Next, we assessed retinal vascular leakage in STZ mice using an FITC-conjugated dextran assay. In STZ mice, extravasated FITC-dextran was pronounced in the entire retinal whole mount, whereas ivP12 treatment markedly mitigated vascular leakage of FITC-dextran in STZ mice **(Figure 2D-E, Figure S5E)**. Pericyte loss is associated with increased vascular permeability and is regarded as a hallmark of early DR. Therefore, we evaluated pericyte coverage of the retinal vessels by immunostaining for the pericyte marker desmin. There was a markedly reduced coverage of desmin^+^ pericytes in C57BL/6J mice after 24 weeks of STZ induction, whereas ivP12 treatment effectively restored desmin^+^ pericyte coverage in STZ mice **(Figure 2F, Figure S5F)**. Moreover, ivP12 attenuated the upregulation of pro-inflammatory cytokines, such as IL-1β, IL-6, MCP-1, and adhesion molecules, including ICAM-1 and VCAM-1, in the retina of STZ mice **(Figure 2G, Figure S3)**. Together, these data demonstrate the protective effect of ivP12 in STZ-induced diabetic mice by ameliorating early DR-associated retinal vascular pathology and inflammation.

### P12 suppressed retinal neovascularization and vascular leakage in the OIR mouse model

**Figure 3:**
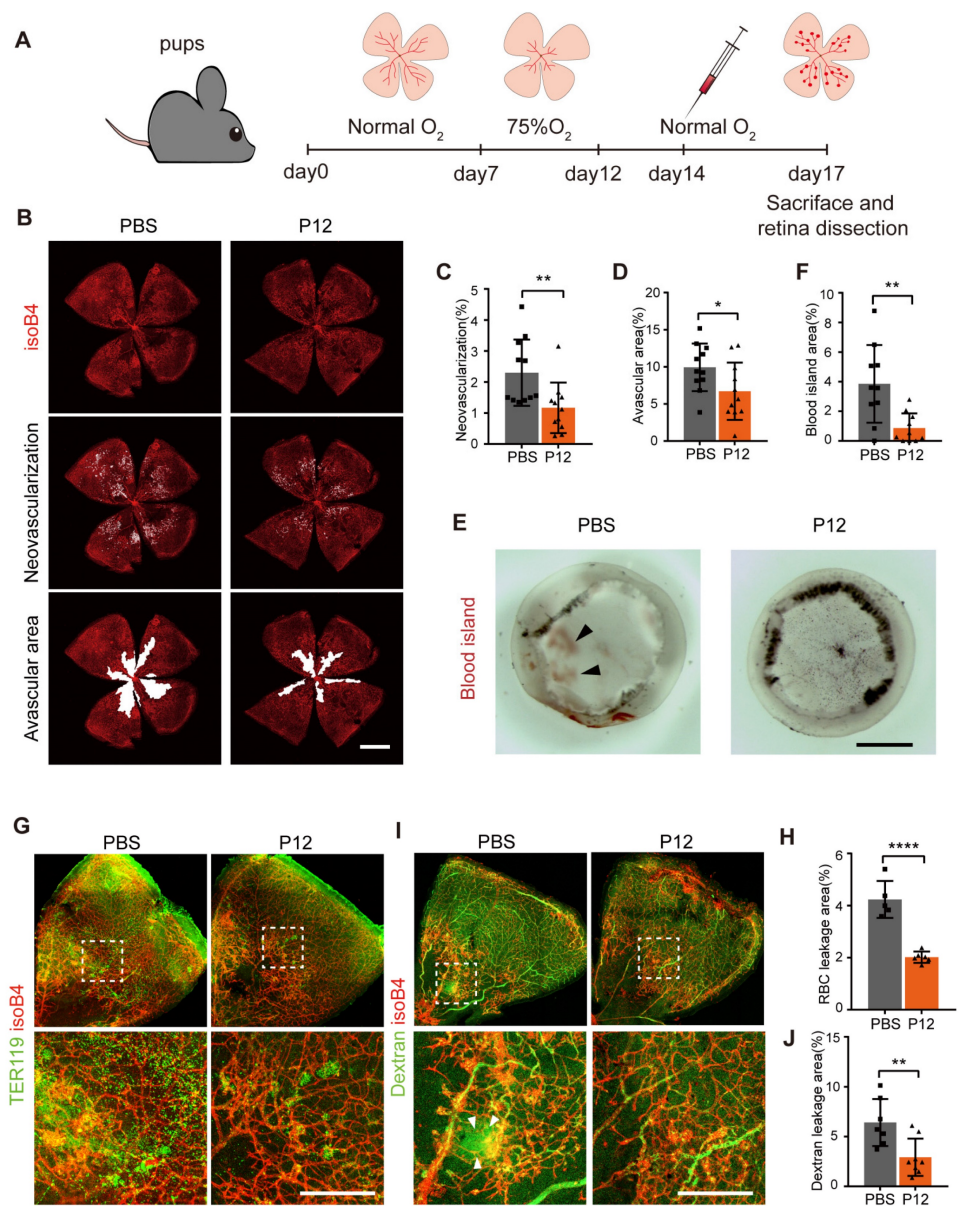
** P12 suppressed retinal neovascularization and vascular leakage in the OIR mouse model.** (A) Schematic depiction of intravitreal P12 treatment in OIR pups. (B-D) P12 markedly decreased the retinal neovascularization and avascular area in the retina of OIR mice. (E) Gross examination of retinas isolated from OIR eye-cups. Black arrowhead depicts the area of vascular leakage (blood island). Scale bar = 500 μm.(F) Quantification of the area of blood island in PBS and P12 treated retinas. (G) Immunostaining of the erythrocyte marker TER119 in retinal flatmount of PBS and P12 treated mice with OIR. (H) Quantification of retinal vascular leakage by calculating the area of extravascular TER119^+^ cells in retinal flatmount. (I, J) P12 markedly decreased the degree of FITC- dextran leakage in the retina of OIR mice. Scale bar = 300 μm.

Since STZ-induced diabetic mice do not fully recapitulate the proliferative vascular phenotype of human disease, we employed an OIR model to investigate the effect of P12 on pathological retinal neovascularization. To induce OIR, mouse pups were exposed to hyperoxic conditions (75% oxygen) from postnatal days 7 to 12 and then returned to room air, as previously described 31 **(Figure 3A)**. We found that the injection of P12, but not the PBS control, on postnatal day 14 significantly reduced retinal neovascularization in OIR pups **(Figure 3B-C, Figure S5A)**. In addition, ivP12 attenuated hyperoxia-induced retinal vascular regression, leading to smaller avascular areas **(Figure 3B-D)**. Pathological neovascularization in OIR mice is associated with an immature and leaky vascular phenotype. Indeed, we observed significant retinal hemorrhage in the eyecups isolated from OIR mice on day 17. In contrast, the area of retinal hemorrhage (defined as a blood island) became smaller or even disappeared in OIR mice treated with ivP12** (Figure 3E-F, Figure S5B)**. Next, we examined vascular leakage by immunostaining for the erythroid marker TER119 and performed an FITC-conjugated dextran assay. We found that the P12-treated OIR retinas showed a reduced area of extravascular TER119 and reduced leakage of FITC-dextran **(Figure 3G-J, Figure S5C-D)**. Overall, these results suggested that ivP12 protects against the development of pathological neovascularization and vascular leakage in OIR mice.

### P12 was mainly engulfed by microglial and endothelial cells in the retina of OIR and STZ mice

**Figure 4:**
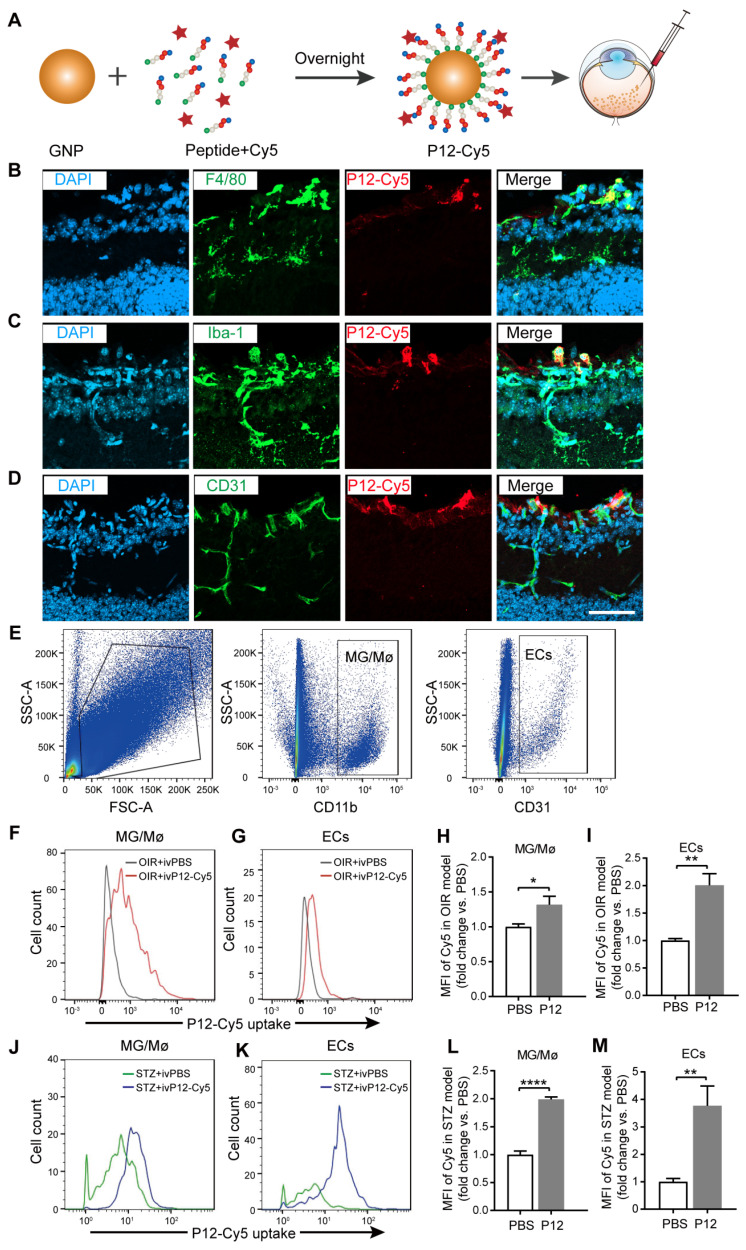
** Identification of P12 targeted cells in the retina of OIR mice and STZ mice.** (A) The construction of Cy5-labelled P12 nanoparticles. (B-D) Co-immunostaining of P12-Cy5 with F4/80 (B), Iba-1 (C), and CD31 (D) revealed colocalization of P12-Cy5 with microglia/macrophage and endothelial cells in the retina of OIR mice. (E) Gating strategy to identify microglia and endothelial cells within the retina of OIR and STZ mouse. (F-I) Uptake of P12 nanoparticle by microglia/macrophage (F, H) and endothelial cells (G, I) in the retina of OIR mice. (J-M) Uptake of P12 nanoparticle by microglia/macrophage (J, L) and endothelial cells (K, M) in the retina of STZ mice. Scale bar = 20 μm.

To identify the target cell types of P12 in OIR and STZ retinas, we fluorescently labeled P12 with Cy5 to visualize its cell-specific uptake in the retina **(Figure 4A)**. The conjugation of P12 with Cy5 does not alter its bioactivity 26. After injecting Cy5-P12 into the vitreous of OIR mice for 24 h, we observed the engulfment of Cy5-P12 mainly by cells in the superficial retinal layer that expressed the microglial markers F4/80 and Iba-1. In addition, we observed the co-localization of P12 with superficial endothelial cells labeled with CD31** (Figure 4B-D)**. To further assess the uptake of P12 by microglia and ECs, we performed flow cytometric analysis by labeling microglia and ECs with CD11b and CD31** (Figure 4E)**. The mean fluorescence intensity (MFI) of Cy5-P12 was calculated to represent the relative quantity of nanoparticles internalized in the microglia or ECs in OIR and STZ mice. In the OIR model, Cy5-P12 or PBS was injected intravitreally into postnatal day 14 pups for 3 days. Compared to the MFI of PBS-treated mice, the MFI was significantly increased in both microglia and ECs in the retinas of P12-Cy5-treated mice **(Figure 4F-I)**. In the STZ model, Cy5-P12 and PBS were intravitreally injected into 24-week STZ-treated mice for 3 days. Similarly, both microglia and ECs showed a markedly increased MFI in P12-Cy5-treated mice compared to PBS-treated mice** (Figure 4J-M).** These results indicate that microglial and endothelial cells are the main cell types that engulf intravitreally injected P12 in the retina.

### P12 inhibited microglia activation and endothelial inflammation partly through NF-kB and MAPK signaling

**Figure 5:**
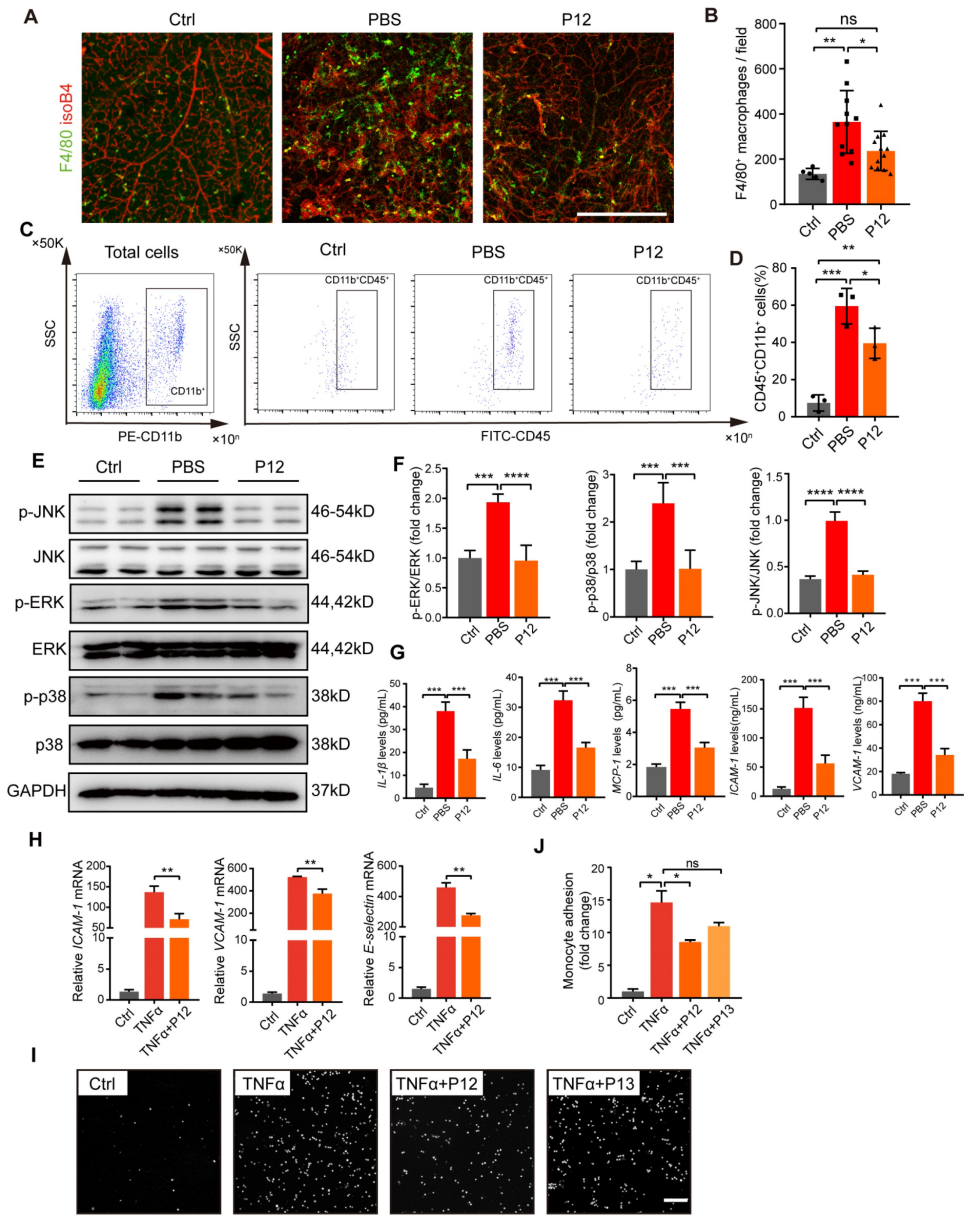
** P12 treatment inhibited microglia activation and endothelial inflammation in the retina of OIR mice.** (A, B) The fluorescence images of OIR retinal tissues revealed the reduced infiltration of F4/80 stained microglial/macrophages under the P12 treatment when compared with the untreated ones. Scale bar = 500 μm. (C, D) Flow cytometry analysis showed reduced levels of CD11b^+^CD45^+^ cells in retina treated with P12 compared to ctrl. (E, F) Western blot and densitometry analysis of the activation of MAPK (p38, JNK, and ERK) signaling in OIR retinas upon PBS or P12 treatment. (G) ELISA measurement of inflammatory cytokines and adhesion molecules in OIR retinas with indicated treatments. The assays shown are representative of at least 3 experiments with similar results, each experiment includes 1 retina sample per group. (H) P12 attenuated TNFα-induced gene expression of adhesion molecules in HUVEC as determined by qPCR. (I) Representative images of fluorescent THP-1 monocytes adherence to HUVEC with the indicated treatments. Scale bar = 100 μm. (J) Adherent THP-1 cells were counted in four randomly selected fields (20× objective) for each treatment group.

Increased inflammatory responses and macrophage infiltration have been reported in the retina of OIR mice. In our study, we observed a significant increase in the number of F4/80^+^ cells in the retinas of OIR mice, indicating increased macrophage infiltration or activated microglial cells. In contrast, P12 treatment significantly reduced the number of F4/80^+^ macrophages/microglia in the OIR retinas **(Figure 5A-B)**. Next, we performed flow cytometry to analyze microglial activation in OIR retinas. Compared to untreated mice, OIR mice showed a marked upregulation of CD45^+^CD11b^+^ cells in the retina, indicating activated microglia. P12 treatment significantly attenuated microglial activation in the OIR retinas** (Figure 5C-D)**. Previous studies have shown that the activation of ERK, p38, and JNK MAPK signaling contributes to DR progression by promoting endothelial inflammation and dysfunction 12, 32. We found that P12 treatment significantly inhibited the activation of MAPK signaling in the retinas of OIR mice** (Figure 5E-F)**. Moreover, ivP12 also markedly suppressed the gene and protein expression of pro-inflammatory factors including IL-1β, IL-6, and MCP-1 and adhesion molecules ICAM-1 and VCAM-1 in the retina of OIR mice **(Figure 5G, Figure S4)**.

To further assess the protective effect of P12 on ECs, we stimulated HUVECs with TNFα to induce endothelial inflammation. We found that the administration of P12 significantly reduced TNFα-induced gene expression of adhesion molecules in HUVECs **(Figure 5H)**. Next, an *in vitro* leukostasis assay was performed on HUVECs to assess the effects of P12 on monocyte adherence to endothelial monolayers. Consistent with the PCR results of adhesion molecule expression, we found that the adhesion of THP-1 cells to HUVECs upon TNFα stimulation was effectively blocked by P12 but not P13 (the inactive control nanohybrid) treatment **(Figure 5I-J)**, indicating that P12 alleviated endothelial inflammation in HUVECs.

To elucidate the downstream signaling pathways of TLR4 regulated by P12, we conducted RNA sequencing analysis on BV2 cells under LPS stimulation with and without P12 or P13 pre-treatment **(Figure S6)**. The heatmap of all differentially expressed genes (DEGs) showed that P12, but not P13, reversed the genetic perturbation in BV2 cells under LPS stimulation (**Figure S6A**). As shown in the Venn diagram, among the genes up-regulated by LPS, 75 genes were reversed by P12 treatment (**Figure S6B**). On the other hand, for the genes down-regulated by LPS, 28 genes were reversed by P12 treatment (**Figure S6C**). Notably, P13 had no effect on the genetic perturbations induced by LPS. The KEGG analysis showed that the MAPK and TNF signaling pathways were the two most significant ones down-regulated by P12 in LPS-stimulated BV2 cells (**Figure S6D**).

**Figure 6:**
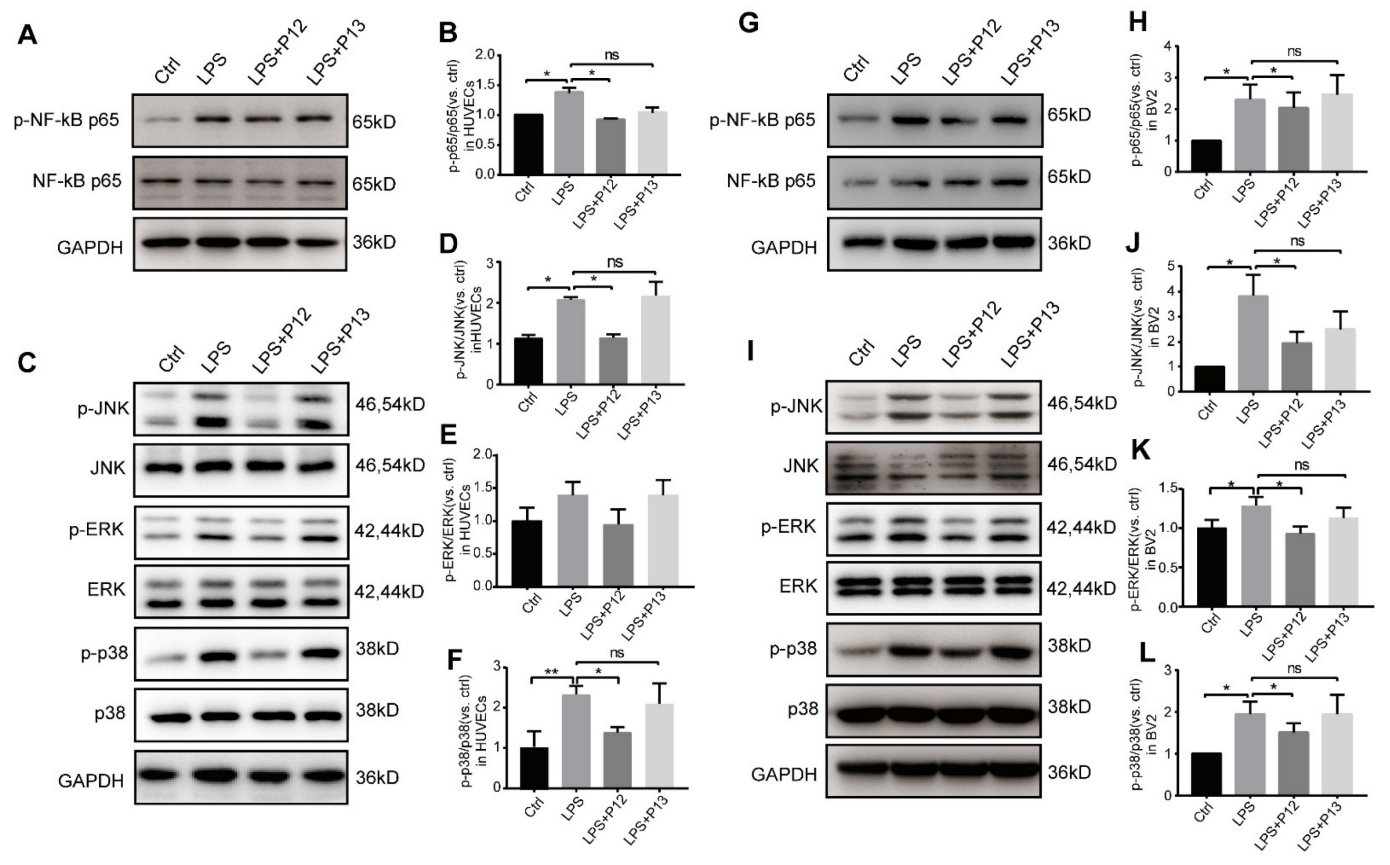
** P12 inhibited the activation of NF-kB and MAPK signaling in HUVECs and BV2 cells.** (A, B) Representative Western blots and densitometry analysis of phospho-NF-kB p65 and NF-kB p65 in HUVECs upon LPS and/or P12 treatment. (C-F) P12 mitigated LPS-induced phosphoactivation of JNK (D), ERK (E), and p38 (F) MAPK signaling in HUVECs. (G, H) Representative Western blots and densitometry analysis of phospho-NF-kB p65 and NF-kB p65 in BV2 cells upon LPS and/or P12 treatment. (I-L) P12 mitigated LPS-induced phosphoactivation of JNK (J), ERK (K), and p38 (L) MAPK signaling in BV2.

Next, we verified these key downstream pathways of TLR4 signaling in HUVECs and BV2 cells. We found that pretreatment with P12, but not P13, significantly inhibited LPS-induced activation of phospho-NF-kB p65 in HUVECs **(Figure 6A-B)** and BV2 cells **(Figure 6G-H)**. Moreover, pretreatment with P12, but not P13, significantly inhibited LPS-induced activation of phospho-JNK and phospho-P38, two classic pro-inflammatory downstream signaling pathways of TLR4, in HUVECs **(Figure 6C-F)** and BV2 cells** (Figure 6I-L)**. Activation of ERK MAPK phosphorylation was observed only in BV2 cells and was inhibited by P12 treatment **(Figure 6I, 6K)**. These findings suggest that P12 may reduce retinal inflammation by inhibiting TLR4 signaling and downstream inflammatory reactions in microglia and endothelial cells.

### P12 exerts its anti-inflammatory effect in HUVECs and BV2 via inhibiting endosome acidification

**Figure 7:**
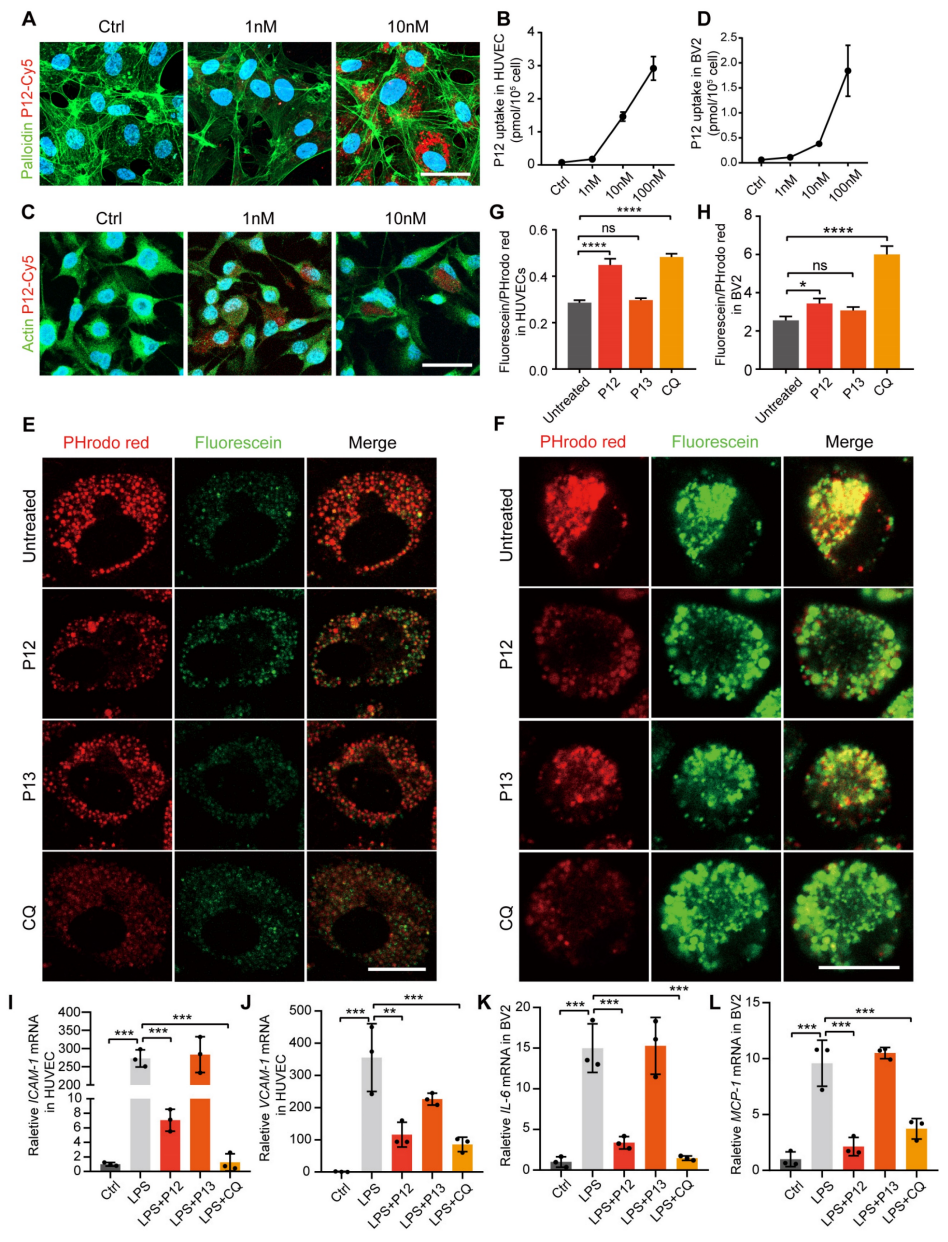
** P12 exerts its anti-inflammatory effect in HUVECs and BV2 cells via inhibiting endosome acidification.** (A) Immunofluorescence image showing the uptake of Cy5-labeled P12 in HUVECs. (B) Dose-dependent uptake of P12-Cy5 in HUVECs. (C) Immunofluorescence image showing the uptake of Cy5-labeled P12 in BV2. (D) Dose-dependent uptake of P12-Cy5 in BV2. (E, F) Confocal images of HUVECs (E) and BV2 (F) cells treated with the nanoparticles and a well-known pH modulator chloroquine (CQ). Endosomal pH was probed with pHrodo red- and fluorescein-labeled dextrans. Scale bar = 20 μm. (G, H) Changes in endosomal pH in HUVECs (G) and BV2 cells (H) were quantified as the fluorescence intensity ratios of green-to-red signals. (I-L) qPCR analysis of inflammatory gene expression in HUVECs (I, J) and BV2 (K, L) cells treated with the nanoparticles and CQ.

Modulation of endosomal pH is crucial for regulating TLR signaling 33, 34. Our previous research showed that phagocytic peptide-GNP hybrids in immune cells can modulate endosome pH and consequently attenuate the signaling of multiple endosomal TLRs 26. To investigate if this also occurs in microglial and endothelial cells, we examined the phagocytic capability of these cells following P12 treatment. We observed that both HUVEC and BV2 cells showed high cellular uptake of Cy5-P12 in a dose-dependent manner **(Figure 7A-D)**. Next, we compared the effect of P12 on endosomal pH with that of chloroquine (CQ), a well-known endosomal pH modulator that inhibits acidification. Endosomal pH was monitored using pH-sensitive fluorescein and pHrodo red dextran. Fluorescein emits strong fluorescence in a neutral environment (pH 6-7.2), whereas the signal of pHrodo red dextran decreases as the pH increases (pH 4-8). As shown in **Figures 7E and F**, the fluorescence signal of pHrodo red dextran was markedly reduced after CQ and P12 treatment compared to the untreated control or P13 treatment. Specifically, P12 treatment, but not P13, increased the fluorescein/pHrodo red intensity ratio from 0.29 to 0.45 in HUVECs **(Figure 7G)** and from 2.55 to 3.44 in BV2 cells** (Figure 7H)**, indicating an elevation in endosomal pH by P12 in these cells. The increase in endosomal PH by P12 is likely attributable to the negative charge of aspartate (with a side chain pKa of ~3.9) on the nanoparticle surface, as demonstrated in our previous study 26.

Next, we assessed the expression of inflammatory factors in HUVEC and BV2 cells to explore the relationship between endosomal acidification and the subsequent activation of the pro-inflammatory response. Our findings demonstrated that both P12 and CQ treatment alleviated the LPS-induced expression of inflammatory factors, including ICAM-1 and VCAM-1 in HUVECs and IL-6 and MCP-1 in BV2 cells **(Figure 7I-L)**. These results suggest that the anti-inflammatory effect of P12 in HUVECs and BV2 cells may be attributed to its ability to inhibit endosomal acidification.

### Toxicity assessment of intravitreal injection of P12

**Figure 8:**
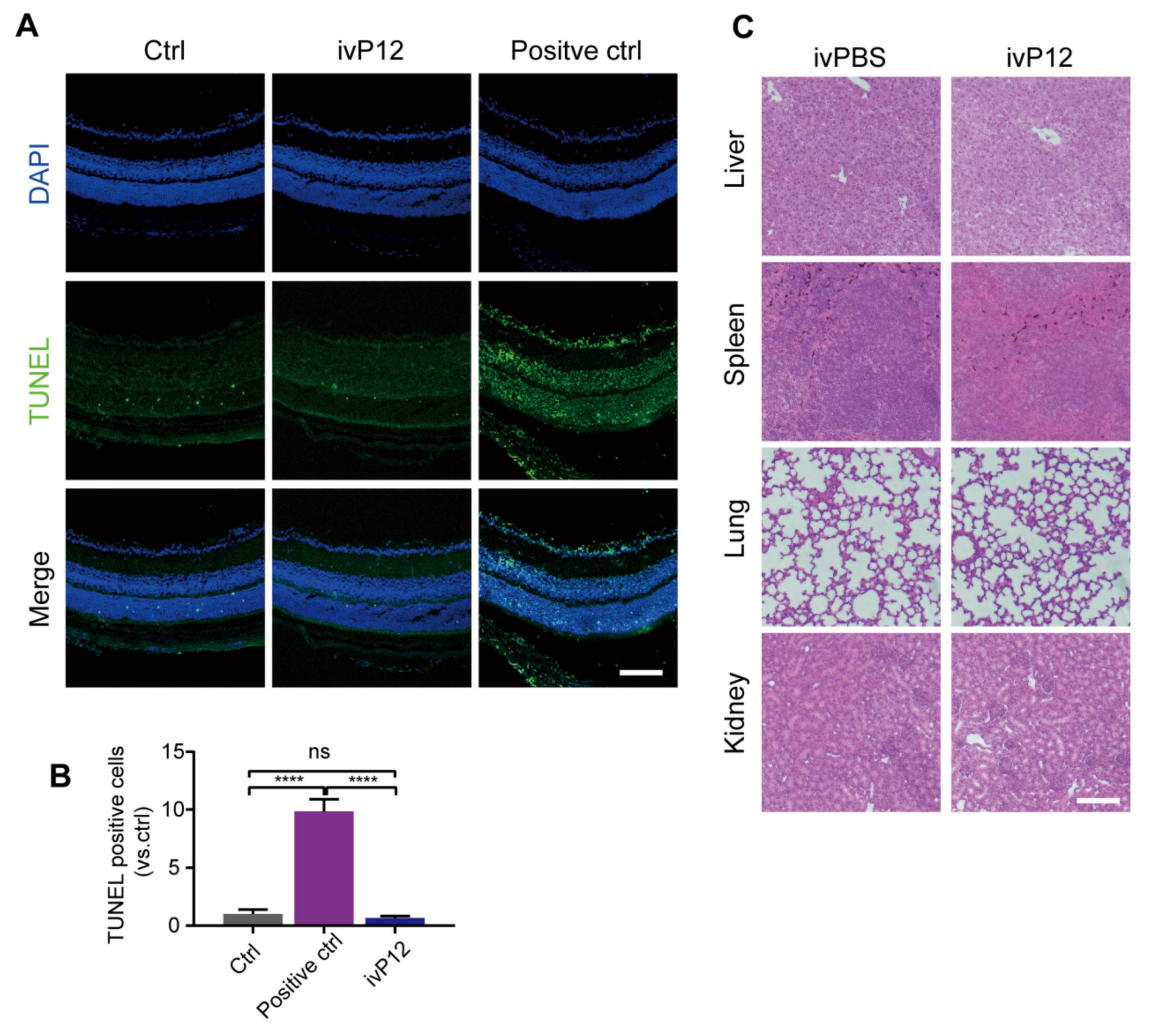
** Toxicity assessment of intravitreal injection of P12.** (A, B) Representative images of TUNEL assay of retinal sections from PBS (Ctrl) or P12-treated mice after 24 h of indicated treatments. Positive control was retinas treated with DNase I. (C) Histopathological analysis of other organs by HE staining in mice intravitreally injected with PBS or P12. Scale bar = 100 μm.

To assess the safety of intravitreal P12 injection, we performed a TUNEL assay to identify apoptotic cells in retinal sections. We found that the number of TUNEL^+^ cells in the retinas of ivP12-treated mice was not significantly different from that in control mice treated with saline **(Figure 8A-B)**. Meanwhile, the retinas of ivP12-treated mice maintained normal histological structure and thickness compared to control mice **(Figure 8A)**. Additionally, we investigated the toxicity of P12 in other major organs, including the liver, spleen, lungs, and kidneys. Histological analysis of these organs revealed relatively intact and normal features in P12-treated mice compared to control mice** (Figure 8C)**, indicating that ivP12 was safe and did not induce obvious toxicity in mice.

## Discussion

The current clinical treatment for DR typically involves long-term, repeated intravitreal injections of anti-VEGF, laser photocoagulation, and vitrectomy. While these methods are commonly utilized and have shown positive outcomes, concerns persist regarding the limited efficacy and potential side effects of medications, as well as the risks associated with major surgical procedures. Nanotechnology has emerged as a promising therapeutic approach in the medical field, offering new possibilities for treating DR 35, 36. We previously developed a novel class of peptide-GNP hybrid-based, drugless bioactive nanodevices that specifically target and attenuate endosomal TLR-mediated immune responses in macrophages 26, 27, 29. In this study, we screened for P12, a compound that effectively targets retinal microglial and endothelial cells to reduce their activation and inflammatory responses. Specifically, P12 treatment successfully inhibited the activation of key transcription factors in the TLR4 signaling pathways, including NF-κB, JNK, and P38-MAPK in microglial and ECs, leading to decreased expression of inflammatory cytokines and adhesion molecules. Importantly, P12 demonstrated efficacy in ameliorating early DR-associated vascular impairments, such as vascular leakage and pericyte loss in STZ-induced diabetic mice, as well as in suppressing pathological neovascularization and retinal hemorrhage in OIR mice. These findings provide promising evidence for the potential use of bioactive peptide-GNP hybrids as next-generation anti-inflammatory agents for DR treatment.

### Bioactive nanodevices offer distinct advantages as promising therapies for the treatment of DR

For many ophthalmic drugs, overcoming the protective barriers of the eye, such as the inner and outer BRB, and effectively delivering drugs to the retina with minimal damage remains a challenge. Nanodrugs exhibit unique physical and chemical properties that make them highly effective therapeutics for various conditions. Their nanoscale sizes (ranging from 0.1 nm to 100 nm) enable them to easily transverse the retinal barriers, such as the BRB 37. Additionally, nanodevices possess a high surface area, making them well-suited for carrying diverse molecular functional motifs for cell targeting, cell penetration, and therapeutic purposes. Furthermore, the size, shape, and surface chemical characteristics of nanodevices can be tailored to meet specific therapeutic requirements 35, making them novel and versatile tools in medicine. Due to these advantages, nanotechnology has been increasingly applied for the prevention and treatment of DR. For example, carbon nanodots have been used to coat two classical anti-VEGF drugs (i.e., bevacizumab and aflibercept) to prevent their degradation. This approach significantly enhances retinal drug concentrations and reduces retinal neovascularization 38.

We previously developed a unique, drug-free nanodevice composed of a GNP core and a hexapeptide coating on the surface 26, 29. The rationally designed peptide coating effectively improved the physiological stability and immunomodulatory properties of GNPs. Through screening, we identified P12 as the most potent anti-inflammatory agent capable of inhibiting a broad spectrum of TLR-mediated inflammatory responses in macrophages and providing significant protection against acute lung injury 26, 27. In the present study, we demonstrated for the first time that intravitreally injected peptide-GNP hybrids effectively reduced retinal inflammation and retinal endothelial impairment in both STZ-induced diabetic and OIR mouse models of neovascularization.

### The drug-free nanodevice P12 targets endosomes of endothelial cells and microglia to modulate TLR4 signaling pathways

TLRs are pattern recognition receptors that recognize numerous pathogenic and danger-associated molecular patterns (PAMP/DAMP). Among TLRs, TLR4 plays a critical role in the development of DR. Numerous studies have shown the upregulation of TLR4 and activation of its downstream signaling cascades in the retina of animal models of DR 19, 21. Additionally, elevated TLR4 expression has been observed in fibrovascular membranes of patients with DR 39. Vagaja *et al.* reported that systemic exposure to LPS in hyperglycemic mice activated TLR4, leading to retinal endothelial injury and central retinal thinning 40. Given the significance of TLR4 signaling in the pathogenesis of DR, targeting the TLR4 pathway could represent a promising therapeutic approach for DR. Indeed, deletion of TLR4 in STZ-induced diabetic mice or OIR mice has shown significant improvements in retinal thinning and pathological neovascularization associated with DR 21, 39, 41. Several TLR inhibitors and antagonists, including small molecules and antibodies, have been developed for clinical use. However, these inhibitors have demonstrated limited therapeutic efficacy in treating inflammatory diseases, such as sepsis.

In this study, we showed that the peptide-GNP hybrid P12 effectively inhibited TLR4 downstream signaling pathways, including the activation of NF-κB, JNK, and P38 MAPK in the retinas of OIR mice, as well as in HUVECs and BV2 cells. The inhibitory effect of P12 on TLR4 signaling is largely attributed to its ability to modulate endosomal pH. Endocytosis and endosome maturation are critical steps in the activation of TLR4 signaling pathways 15. Activated TLR4 is initially targeted to early endosomes (pH 6.5), then transferred to late endosomes or multivesicular bodies (pH 4.5) before being recycled to the cell surface or degenerated 42. Specifically, the MyD88-dependent pathway primarily occurs at the plasma membrane, whereas the MyD88-independent (or TRIF-dependent) pathway is activated when the TLR4 ligand, LPS, is internalized and binds to TLR4 within endosomes. Our previous study showed that a specific peptide coating on P12 enhances endosomal uptake 27. Once accumulated in endosomes, P12 disrupts normal endosomal acidification, likely due to the “buffering effect” of the negative charges of aspartate on the nanodevice surface, thereby inhibiting the downstream TLR4 signaling. Our findings revealed that P12 significantly increased endosomal pH in both HUVECs and BV2 cells. The pH-modulating effect of P12 was comparable to that of CQ, a weak base that can rapidly enter endosomes and elevate their pH. Furthermore, treatment with P12 or CQ significantly suppressed LPS-induced inflammatory gene expression, indicating a connection between altered endosomal acidification by P12 and the subsequent inhibition of pro-inflammatory responses.

### The retinal distribution of peptide-GNP hybrids after intravitreal vs. periocular injection

This study examined the optimal retinal delivery route and biodistribution of the peptide-gold nanoparticle hybrids. Intravitreal, periocular, subretinal, and systemic routes are the four commonly used routes for retinal drug delivery, each with its advantages and drawbacks 35. Intravitreal injections are the most commonly used in clinical practice, ensuring direct drug delivery to the retina. However, this method is invasive and may cause adverse complications, especially with repeated use. Periocular injection offers a minimally invasive alternative for retinal drug delivery, although drug distribution in the retina is relatively inadequate. To determine the ideal route for delivering peptide-GNP hybrids to the retina, we compared the efficacy of P12 delivery via intravitreal and periocular injections. 6 h after injection, we found that Cy5-labeled P12 was more abundant in the intravitreal-injected eyes than in the periocular-injected eyes, suggesting that the intravitreal route was more efficient for retinal P12 delivery. Moreover, TEM analysis showed that 6 h after the intravitreal injection, P12 was mainly observed in the nerve fiber layer of the retina, which consists mainly of superficial blood vessels and microglia.

Immunohistochemistry and flow cytometry revealed that ivP12 was mainly engulfed by microglial cells, which are the major phagocytic cells in the retina. Surprisingly, retinal endothelial cells also exhibited high uptake of P12 in both *in vitro* and *in vivo* experiments. Previous reports suggest that the uptake of nano-drugs by endothelial cells may be triggered by caveolin-mediated endocytosis 43, 44. Given that vascular impairment is a primary pathological feature of DR, biologics that specifically target retinal endothelial cells could provide additional benefits by directly addressing early vascular defects, thereby preventing severe vascular abnormalities. Thus, intravitreal injection of P12 may be the preferred treatment approach for DR, ensuring optimal drug biodistribution within retinal blood vessels.

## Conclusions

In conclusion, this study demonstrated that intravitreal injection of the drug-free, peptide-GNP hybrid P12 significantly improved early DR symptoms, including vascular leakage and pericyte loss, in diabetic mice. P12 also suppressed pathological neovascularization and retinal hemorrhage in the OIR mouse models. The intravitreally injected P12 was mainly taken up by microglial and endothelial cells, leading to reduced retinal endothelium inflammation and microglial activation in DR animal models. P12 potently inhibited several TLR4 downstream signaling pathways, such as the activation of NF-κB, JNK, and P38 MAPK, in both endothelial and microglial cells. Such effects are likely due to its capacity to block the endosome acidification process. This novel, drug-free, bioactive nanoparticles-based therapeutic approach represents a promising alternative to overcome the limitations of traditional intravitreal interventions, providing efficient and sustained treatments to the retina.

## Methods

### Chemicals and reagents

The TLR agonists LPS (#L3204, from Escherichia coli serotype) was purchased from Sigma-Aldrich (Darmstadt, Germany). The antibodies against-GAPDH (#UM4002) was purchased from Utibody, antibody against NF-κB p65 (#8242) and phospho-NF-κB p65 (#3033), p44/42 MAPK (Erk1/2) (#9102S) and phospho-p44/42 MAPK (Erk1/2) (#4370S), p38 MAPK (#8690) and phospho-p38 MAPK (#4631), JNK (#9258S) and phospho-JNK (#4668S) were obtained from Cell Signaling Technology (Boston, MA, USA). Fluorochrome-labeled antibodies against mouse CD45 (#103108), CD31 (#102405), CD11b (#101207), and CD284 (TLR4) (#145406) were from BioLegend (San Diego, CA, USA). We obtained the primary antibodies against CD31 (#557355) from BD Biosciences (San Diego, CA, USA), F4/80 (#30325S) from Cell Signaling Technology (Boston, MA, USA), iba-1 (#019-19741) from Wako, TER119 (#MAB1125) from R&D Systems (Minneapolis, MN, USA). Isolectin B4 Alexa Fluor 594 (#I21413) was obtained from Jackson immune Reseach (USA) and dextran (20kD, #FD70S; 3kD, #FD4) from Sigma-Aldrich (Darmstadt, Germany). Anti-Desmin-Cytoskeleton Marker (ab32362) were purchased from Abcam (USA). The FragELTM DNA Fragmentation Detection Kit (QIA39) was obtained from Millipore (USA).

### Cell culture

Human Umbilical Vascular Endothelial Cells (HUVECs) were cultured in endothelial cell growth medium (ECGM) containing 5% FBS, 1% endothelial cell growth factor and 1% antibiotic solution and were used from passage 2 to passage 6. BV2 cell line was purchased from Kunming Cell Bank, and was cultured in DMEM cell medium containing 10% FBS and 1% antibiotic solution. THP-1 cells were purchased from the Cell Bank of Chinese Academy of Sciences, and were cultured in DF-12 cell medium containing 10% FBS and 1% antibiotic solution. For stimulation experiments, cells were starved 3 h in cell medium supplemented with 2% FBS and 1% antibiotic solution.

### Synthesis and physicochemical characterization of peptide-GNP hybrids

GNPs were synthesized according to a modified procedure from the literature and our earlier work. The fabrication of peptide-GNP hybrids was conducted following our published protocol 45. Briefly, hexapeptides (CanPeptide Inc., Montreal, Canada) were dissolved in endotoxin free, ultrapure water as a stock solution of 1 × 10^3^ M. Two types of hybrids (P12 and the inactive control P13) were made by mixing ten volumes of the synthesized GNP solution (final concentration of GNPs is 11 × 10^9^ M) with one volume of peptide stock solutions. For fluorescent nanoparticles, one volume of peptide stock solution (1 × 10^3^ M) containing 0.5% Cy5-PEG5000-SH (1 × 10^3^ M in PBS, Nanocs Inc. New York, USA) was mixed with ten volumes of synthesized GNP solution. After overnight incubation, the hybrids were filtered through a syringe filter (0.22 µm, Milipore, Billerica, MA, USA), followed by centrifugation (18 000 g at 4°C for 30 min). The pellets were then washed three times with sterile PBS (HyClone, GE Healthcare) to remove unbound peptides, and resuspended in PBS or cell culture medium to have a final concentration of 1 × 10^6^ M prior to use.

The size and morphology of the bare GNPs and hybrids were visualized using a Tecnai Osiris scanning TEM (FEI, Hillsboro, USA) at an accelerating voltage of 200 kV. The average hydrodynamic diameter of the bare GNPs and hybrids were determined by DLS on a Zetasizer (Malvern Instruments, Worcestershire, UK). The quality of the GNPs was assessed by their absorption spectrum collected on a spectrophotometer (Lambda 35 UV-Vis Spectrometer, Perkin Elmer Inc, USA). To further evaluate the stability of the hybrids in salt solutions, 50 µl of P12 or bare GNP (10 × 10^9^ M) were mixed with equal volume of NaCl solutions with various concentrations ranging from 0.3 to 3 M in a 96-well plate and incubated for 2 h at room temperature. The optical density (OD) at 524 nm was measured with a plate reader (Varioskan Flash, Thermo Scientific, Waltham, USA). The data were normalized with the OD value in the absence of NaCl.

### Streptozocin (STZ) induced diabetic mouse model

To induce the type I diabetic mouse model, STZ (150 mg/kg) was intraperitoneally injected into C57BL/6J mice. The blood glucose and weight levels were measured monthly to confirm the successful induction of diabetes. P12 (500 nM, 1 uL) was injected intravitreally into C57BL/6J mice at 7 days, 1 month and 5 months after STZ injection. Retina samples were collected at 6 months after STZ-induced diabetes for analysis. All study protocols involving the use of animals were approved by the Institutional Animal Care and Use Committee of Tianjin Medical University (TMUaMEC 2020006).

### Oxygen-induced retinopathy (OIR) mouse model

Oxygen induced retinopathy model was performed as previously described 31. Briefly, Pups on the 7^th^ day after birth with the nursing mother were exposed to hyperoxia (75% O2). After 5 days, the mice were taken back to room air. For applying treatment, P12 (500 nM, 0.5 uL) was injected intravitreally at the 14^th^ day after birth. The retinas were collected at P17 for analysis.

### Immunofluorescence

Retina dissections were obtained as previously described 31. Briefly, for flat-mounted retinas, eyes were enucleated from mice and fixed in 4% PFA for 1 h at room temperature. Retinas were dissected, washed with PBS containing 1% TritonX-100 overnight at 4°C then blocked in PBS containing 2% BSA, 0.3% TritonX-100 for 12 h at 4°C. To observe the retinal vasculature in OIR models, the flat-mounts were stained by isoB4 (1:200) for 2 h at RT. For staining the RBC leakage and macrophages infiltration in the OIR models, the blood vessels and the pericytes in the STZ models, rat anti-TER119 monoclonal (1:200); rabbit anti-F4/80 monoclonal (1:200); rat anti-CD31 monoclonal (1:200); anti-Desmin (1:100) antibodies were incubated in blocking solution at 4°C overnight. After washing, the retinas were incubated with corresponding secondary antibodies (1:300, Jackson Immuno Research, US) for 2 h at RT. Flat-mounted retinas were analyzed using a confocal fluorescence microscope (LSM 900, Carl Zeiss, Germany).

For retina frozen sections, the eyes were fixed in 4% PFA for 1 h at room temperature, then gradient dehydrated in 10%, 20%, 30% saccharose solution at 4°C and subsequently embedded in optimal cutting temperature compound (OCT) and frozen at -80°C. Then sagittal sections of the eyes were prepared and store at -80°C. The sections were rewarming at RT for 30min, then residual OCT was washed 3× with PBS. Similar to the flat-mount, the retina sections were blocked in PBS containing 2% BSA, 0.3% TritonX-100 for 1 h at RT, then certain antibodies, mouse anti-iba1 monoclonal (1:200); rat anti-CD31 monoclonal (1:200); rabbit anti-F4/80 monoclonal (1:200) were incubated in blocking solution at RT for 2 h. After washing, the sections were incubated with corresponding secondary antibodies at RT for 1 h. Then the sections were mounted with sealing agent with DAPI to store and observe the distribution of P12-Cy5 in retinas.

### Analyses of vascular leakage and perfusion in OIR and STZ mice

Vascular leakage was analyzed by intracardial injection performed as previously described 46. In brief, 31-G needle was positioned above the heart 2 mm parasternal to the left at a virtual line connecting both armpits, in caudal and lateral angles of 30° and 10°, 50 μL, warm PBS containing FITC-conjugated dextran were injected. In OIR model, the molecular weight of dextran was 20kD, after 10 min of circulation, eyes were enucleated and fixed in 4% PFA for 1 h at 4°C. Retinas were then dissected, washed with PBS and permeabilized with PBS containing 1% TritonX-100 overnight at 4°C and stained with immunofluorescence. In STZ models, the molecular weight of dextran was 3kD, after 10 min of circulation, the mice were perfused with 15-20mL warm PBS for 3 min to remove the residual dextran in vessels. The retina was dissected and fixed for analysis. For the dextran permeation qualification, the plasma was obtained before the perfusion of STZ models, then after perfusion, the retinas were dissected and weighted, then rubbed in ice cold PBS, after centrifugation (15000g×20min, 4°C), the supernatant was kept. The intensity of dextran in the plasma and supernatant of retina were measured by spectrophotometer at 490/530 nm. The dextran permeation degree was calculated by following formula:

Dextran permeation degree (mL/g) = 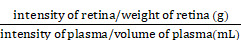


### Ocular distribution of P12

For Living Image Spectrum, P12-Cy5 were administered retro-orbitally (500nM, 20uL) or intravitreally (500 nM,1 uL) into CD1 mice. After 12 h,eyes were harvested for imaging with IVIS spectrum (PE, Waltham, MA, US). The fluorescence intensity was measured by using the ROI tool of Living Image software (V4.2). Briefly, the region of the eyes was selected by using the ROI tool and the fluorescence intensity was measured.

For Transmission Electron Microscope, P12 were administered retro-orbitally (500 nM, 20 uL) or intravitreally (500 nM,1 uL) into C57BL/6J mice. After indicated time points, eyes were enucleated and fixed in 2.5% glutaraldehyde (Solarbio) for 2-4 h. Then the cornea and len was removed and the posterior eye cup was dissected, cut into small 1 mm^3^ clumps, post-fixed with 1% osmium tetroxide in a 0.1 M phosphate buffer (PB, pH 7.4) at room temperature for 2 h, dehydrated in a graded ethyl alcohol series, and embedded in SPI-Pon 812 epoxy resin (SPI, Cat#90529-77-4, PA, USA) overnight at 37°C. The embedded tissues were polymerized for more than 48 h at 60°C. Then 60-80 nm ultra-thin sections were cut using a Leica UC7 ultramicrotome, stained with 2% uranium acetate saturated alcohol solution for 8 min and 2.6% lead citrate for 8 min, and observed under an HT7800 transmission electron microscope (HITACHI, Tokyo, Japan).

### P12 targeted retinal cell types

For flow cytometry, retina dissection was performed as previously described 31. Briefly, P12-Cy5 was intravitreal injected to the STZ and OIR mice, after 24 h, the retinas were dissected and washed with ice cold PBS, then were transfered to the enzyme mix solution, for 4-5 retinas, 1.5mg Collagenase/Dispase and 0.125mg DNase I were added to 500uL endothelial cell growth medium containing 5% FBS and 1% antibiotic solution, then the retina tissue were clipped into little pieces, and digested into cell suspension with gently rotating in tissue mixer for 20 min, 37℃.

After washing 3× with PBS and centrifugation (4500rpm at 4°C for 5 min), retina tissue were filtrated by strainer (d=70 um) to obtain the single cell suspension. PE-CD11b and FITC-CD31 antibodies (1:200) were used to mark the microglia and ECs. The data were collected on a LSRII flow cytometer (BD Biosciences), and the quantity of P12 was analyzed by the fluorescence intensity of Cy5 in certain cell types.

### Endosome acidification assay

HUVECs or BV2 cells were seeded in con-focal dishes (d=15 mm) to adherence overnight, then were incubated for 6 h with a mixture of fluorescein- and pHrodo red-labeled 10,000 MW dextrans (10 mg/ml) (Life Technologies). Cells were then washed with PBS three times, and treated with chloroquine (50 mM), P12 (100 nM) or P13 (100 nM) for 1 h. Cells were then washed with PBS three times and imaged by confocal microscope (Zeiss LSM900, Germany). The intensities of Fluorescein and pHrodo red fluorescence within intracellular vesicles were quantified, and the ratio of fluorescein to pHrodo red fluorescence was calculated. A minimum of 20 cells were qualified in each group for three independent experiments.

### Monocyte adhesion

HUVECs were seeded in six well plates to adherence overnight. Cells were treated in serum-starved 2% FBS medium with BSA or TNF-α (10 ng/mL) for 6 h with a 2 h preincubation with P12 or P13. THP-1 monocytes were counted and cell suspension of THP-1 was incubated with BCECF fluorochrome for 30 min, 1×10^6 cells per well were seeded and co-cultured with HUVECs for 3 h. Unbound THP-1 monocytes were thoroughly removed by gently washing 3× with PBS, and cells were fixed in 4% PFA for 15 min. Adherent monocytes were photographed at fluorescence 488 using microscope at a magnification of 20×, and were counted in at least four random fields for each treatment.

### TLR4 downstream signaling analysis

To detect the TLR4 downstream signaling, the HUVECs and BV2 cell were pretreated with P12 or P13 for 2 h, then stimulated with LPS (1 ug/mL) for 30 min. Cells were washed with ice cold PBS and lysed in cold RIPA lysis buffer supplemented with 1× protease inhibitor cocktail and 1× phosphatase inhibitor (Roche, Switzerland). Proteins (30 μg for each sample) were subjected to 10% SDS-polyacrylamide gel electrophoresis and transferred to a polyvinylidene difluoride membrane (Millipore, Germany). Target proteins were detected by specific primary antibodies against JNK and p-JNK (phospho-Thr183/Tyr185), p38 and p-p38 (phosphor-Thr180/Tyr182), ERK and p-ERK (phosphor-Thr202/Tyr204), NF-κB p65 and p-NF-κB p65(phosphor-Ser536). GAPDH was used as loading control. Then the banded primary antibodies were detected by the indicated second antibodies. The intensity of proteins were qualified by ECL western-bolting systems, and analyzed via imageJ software. Band intensities were normalized to GAPDH.

### RNA extraction and quantitative real-time PCR analysis

Retinas were dissected and homogenized in TRIzol® reagent (Invitrogen, US). RNA samples were reverse-transcribed to complementary DNA (cDNA) using TransScript One-Step gDNA Removal and cDNA Synthesis SuperMix (TransGen, China). qPCR was performed using PerfectStartTM Green qPCR SuperMix (TransGen, China) and was processed with QuantStudio 5 Real-Time PCR system (Applied Biosystems, US). β-actin was used as internal control. The primers used in this study were: β-actin forward, 5'-GGCTGTATTCCCCTCCATCG-3'; β-actin reverse, 5'-CCAGTTGGTAACAATGCCATGT-3'; Il1β forward, 5'-GCAACTGTTCCTGAACTCAACT-3'; Il1β reverse, 5'-ATCTTTTGGGGTCCGTCAACT-3'; Il6 forward, 5'-CCAAGAGGTGAGTGCTTCCC-3'; Il6 reverse, 5'-CTGTTGTTCAGACTCTCTCCCT-3'. Icam1 forward, 5'-GTGATGCTCAGGTATCCATCCA-3'; Icam1 reverse, 5'-CACAGTTCTCAAAGCACAGCG-3'; Vcam1 forward, 5'-AGTTGGGGATTCGGTTGTTCT-3'; Vcam1 reverse, 5'-CCCCTCATTCCTTACCACCC-3'; Mcp1 forward, 5'-TTAAAAACCTGGATCGGAACCAA-3'; Mcp1 reverse, 5'-GCATTAGCTTCAGATTTACGGGT-3'.

### Enzyme-linked immunosorbent assay (ELISA)

To measure the proteins levels of proinflammatory cytokines, the following ELISA kits were used according to the manufacturer's protocol (Jiangsu Jingmei Biological Technology Co., Ltd., China): IL-1β (catalog no. JM-02323M2), IL-6 (catalog no. JM-02446M2), ICAM-1 (catalog no. JM-02466M2), VCAM-1 (catalog no. JM-02412M2), and MCP-1 (catalog no. JM-02365M2). For OIR mice, retina sample were collected from OIR pups treated with or without P12 at postnatal day 17. For STZ mice, the mice received PBS or P12 (500 nM, 1 µL) treatment one week after STZ injection, and retinal samples were collected one month after diabetes induction.

### RNA library preparation and sequencing

For RNA extraction, BV2 cells were cultured in 6-well plates until 80% confluency. Following serum starvation, cells were pretreated with the nanohybrids P12 or P13 for 2 h. Subsequently, BV2 cells were stimulated with LPS for 30 min, while HUVEC cells were stimulated with LPS for 2 h. RNA was then extracted from the cells, and subsequent sequencing techniques were conducted with support from Novogene. For bulk RNA-seq analysis, the differentially expressed genes were determined by DESeq2 package (version 1.42.1) in R (version 4.3.3). P.adjust < 0.05 and |log2(fold change)| > 0.5 were defined as the thresholds for significant differential expression in the heat maps and venn diagrams. Further KEGG analysis were conducted by clusterProfiler package in R. The pathways with p value < 0.05 and q value < 0.05 were regarded as significantly enriched.

### Toxicity assay

Toxicity assessment was performed at wild type mice. Terminal deoxynucleotidyl transferase mediated dUTP nick end labeling (TUNEL) assay was used to examine apoptotic cells in retinas. Mice were sacrificed after 12 h with or without P12 treatment. The eyes were fixed and sectioned as previous described, and store at -80°C. TUNEL assay was performed using a FragELTM DNA Fragmentation Detection Kit according to the manufacturer's instructions.

H&E staining was used to examine the morphology of overall major organs, including liver, spleen, lung and kidney from mice with or without P12 treatment. Organs were fixed with 4% formaldehyde overnight at RT, embedded in paraffin, and sectioned. Images were obtained using a microscope (Nikon, Japan).

### Statistical analysis

Results were expressed as the mean ± SEM. To calculate statistical significance, the Mann-Whitney test or student's t test (two-tailed) were used when comparing two groups, and one-way ANOVA followed by Tukey's multiple comparisons test was used when comparing three or more groups. P < 0.05 were considered significant. All calculations were performed using Prism software.

## Supplementary Material

Supplementary figures.
