# Supplementary material for: Drugless peptide-based nanohybrids alleviate diabetic retinopathy by suppressing microglial activation and endothelial inflammation
Source: Theranostics. 2025 Mar 3;15(9):3943–60. doi: 10.7150/thno.102775 (PMC11980661; doi:10.7150/thno.102775)
Supplement: Supplementary file 1 — Supplementary figures. [file thnov15p3943s1.pdf]

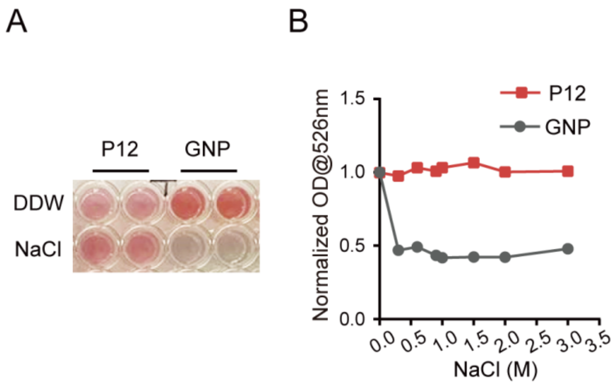

Supplemental Figure S1. Modifying the GNPs with the peptides significantly enhanced their physiological stability and altered their surface properties. (A) Stability of P12 was better than bare GNP in NaCl solutions. (B) Quantification of the optical density (OD) at 524 nm of P12 and bare GNP in NaCl solutions with various concentrations ranging from 0.3 to 3 M.

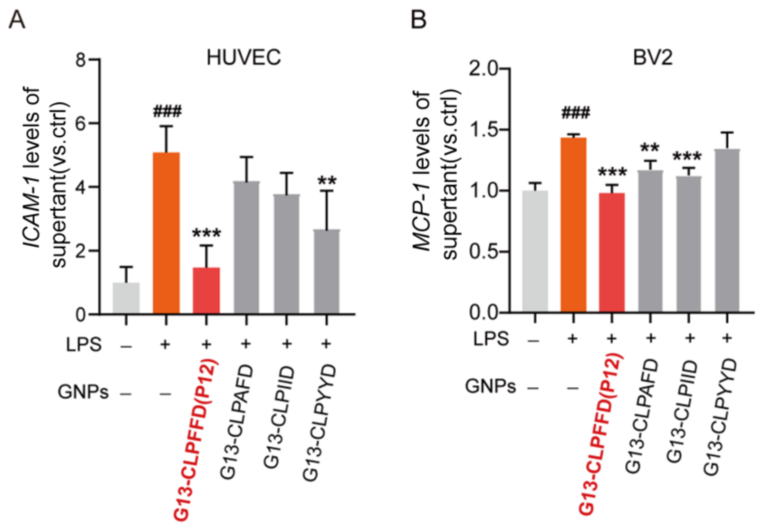

Supplemental Figure S2. ELISA measurement of ICAM-1 and MCP-1 levels in the supernatant of HUVEC (A) and BV2 (B) cells with various peptide-GNP hybrids treatment.

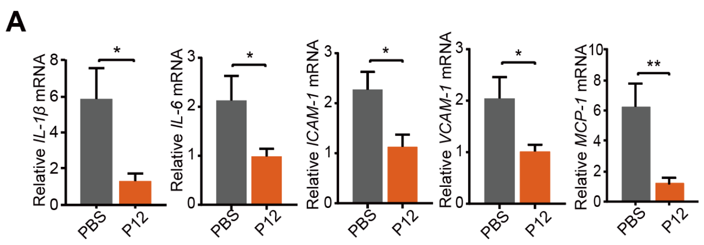

Supplemental Figure S3. P12 attenuated the gene expression of proinflammatory factors in the retina of STZ mice.

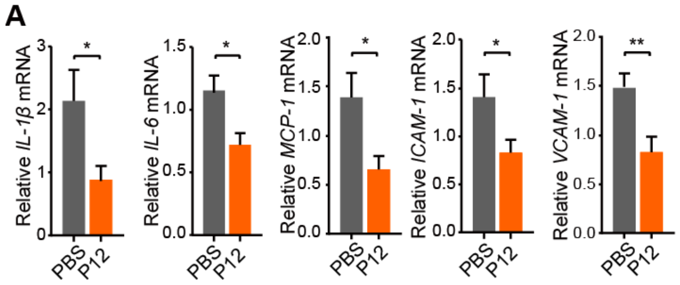

Supplemental Figure S4. P12 attenuated the gene expression of inflammatory cytokines and adhesion molecules in OIR retinas.

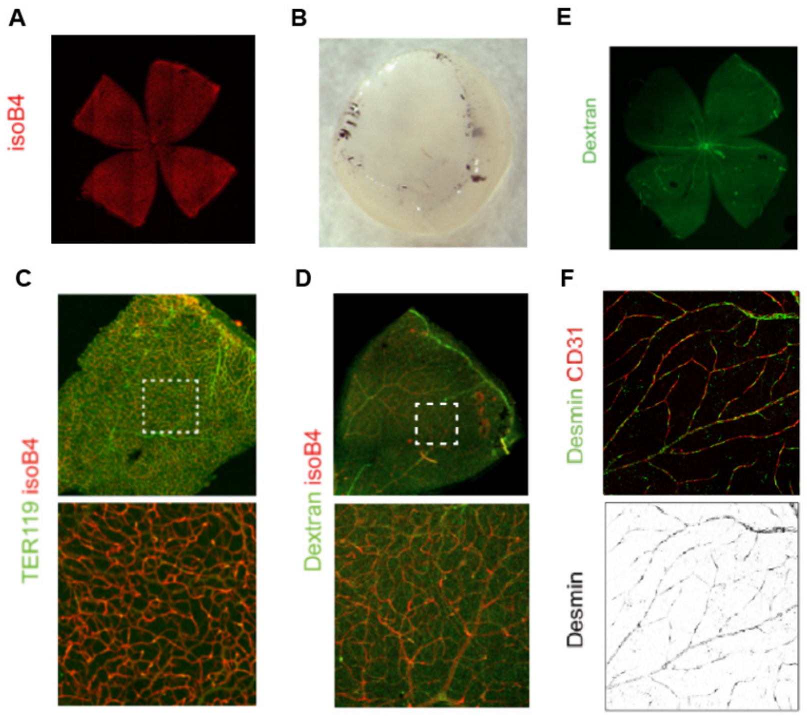

Supplemental Figure S5. (A) Immunostaining of isoB4 in retinal wholemount from postnatal day 17 healthy pups. (B) Eyecups isolated from postnatal day 17 healthy pups. (C) Immunostaining of TER119 in retinal wholemount of postnatal day 17 healthy pups. (D) Injection of FITC-dextran into the retina of healthy mice at postnatal day 17. (E) Assessment of retinal vascular permeability by FITC-dextran assay in 24-week-old wild-type mice. (F) Immunostaining of pericyte marker desmin in retinal wholemounts of 24-week-old wild-type mice.

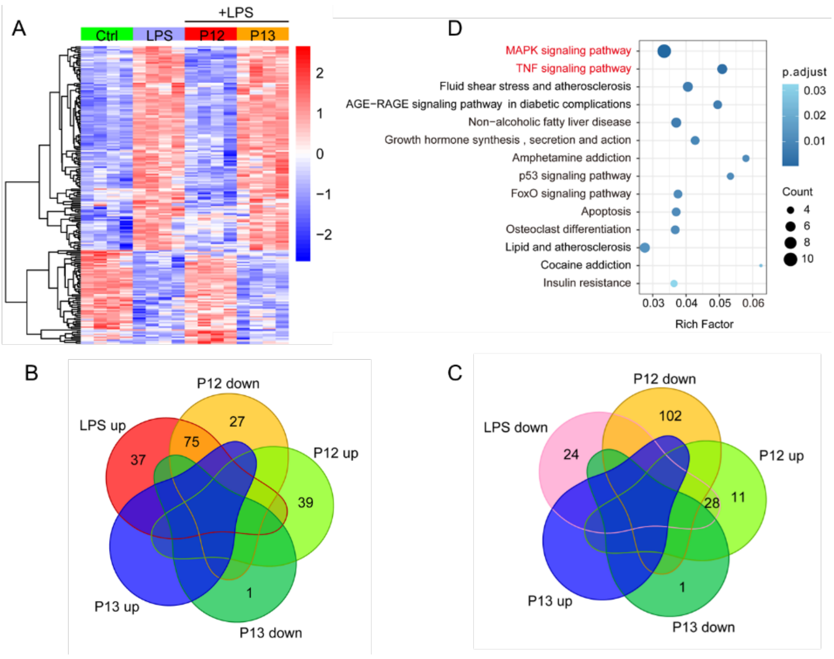

Supplemental Figure S6. The effects of P12 on the LPS-induced transcriptomic alteration by RNA-Seq analysis on BV2 cells. (A) The heatmap of DEGs in BV2 cells affected by P12 or P13 upon LPS stimulation. Each experiment was conducted in four biological repeats. padj < 0.05, log2|FC| > 0.5. (B, C) Venn diagram showing the LPS up-regulated (B) and down-regulated (C) DEGs affected by P12 or P13 treatment. (D) KEGG analysis showing the major down-regulated pathways by P12 in LPS-treated BV2. P value cutoff=0.05, q value cutoff = 0.05. LPS = 1 μg/mL, P12 and P13 = 10 nM.
